# *Mestus cruciatus*, a new delphacid species from southwest China with some remarks on the genus (Hemiptera, Fulgoromorpha, Delphacidae)

**DOI:** 10.3897/zookeys.545.5992

**Published:** 2015-12-14

**Authors:** Feng-juan Ren, Qi Xie, Dao-zheng Qin

**Affiliations:** 1 Key Laboratory of Plant Protection Resources and Pest Management of Ministry of Education, Entomological Museum, Northwest A&F University, Yangling, Shaanxi 712100, China; 2 Baoji University of Art and Science, Baoji, Shaanxi 721013, China

**Keywords:** Auchenorrhyncha, Fulgoroidea, planthopper, taxonomy, distribution, new species

## Abstract

A new delphacid (Hemiptera, Fulgoromorpha, Delphacidae) species, *Mestus cruciatus* **sp. n.** is described based on specimens from Yunnan Province, China. Habitus photos and illustrations of male genitalia are provided. The *Mestus* species and phylogenetic arrangement of this genus is discussed. A key to the species of *Mestus* is also provided.

## Introduction

The Oriental planthopper genus *Mestus* was established by Motschulsky (1863) with *Mestus morio* as the type species from Sri Lanka. It is a small genus in the Delphacini of Delphacinae with two species currently recognized, *Mestus morio* Motschulsky and *Mestus tungpuensis* Yang (Motschulsky 1863; Metcalf 1943; Fennah 1973–75; Yang 1989). It is distributed in Sri Lanka, the Philippines and south China (Taiwan). Recent identification of material in the collections of NWAFU has led to the discovery of a new species of this genus from Yunnan Province (southwest China) and it is described here.

## Materials and methods

The specimens examined in this study including type material are deposited in the Entomological Museum, Northwest A&F University, Yangling, Shaanxi, China (NWAFU). The genital segments of the examined specimens were macerated in 10% KOH and drawn from preparations in glycerin jelly with the aid of a light microscope. Line diagrams were drawn using an OLYMPUS PM-10AD microscope. Photographs were taken with an automontage QIMAGING Retiga 4000R digital camera (CCD) stereozoom microscope. The terminology in this paper follows that of Ding (2006). Measurements of the body length were from the apex of the vertex to the posterior tip of the abdomen. All measurements are in millimeters (mm).

## Taxonomy

### Genus Mestus

Taxon classificationAnimaliaHemipteraDelphacidae

Motschulsky, 1863

Mestus Motschulsky, 1863: 111; Distant 1906: 489; Fennah 1973–75: 85; Yang 1989: 161; Ding 2006: 396.

#### Type species:

*Mestus morio* Motschulsky, 1863 by original designation.

#### Diagnosis.

The genus *Mestus* Motschulsky is readily separated from other genera in the Delphacini of Delphacinae by the vertex with apices of submedian carinae feebly developed, by the median frontal carina distinct but feeble at base, by the post-tibial spur without teeth along posterior margin, by the caudal margin of pygofer strongly produced near base, by the pygofer with a single process on the midventral margin, and by the aedeagus with teeth subapically on both sides.

#### Description.

Head including eyes nearly as wide as pronotum. Vertex quadrate, anterior margin rounded, apices of submedian carinae and base of median frontal carina feebly developed. Angle of fastigium obtuse. Y-shaped carina with common stem distinct. Antennae cylindrical, short. Spinal formula of hind leg 5-7-4, post-tibial spur cultrate, concave on inner surface without teeth along posterior margin. Male pygofer in profile wider ventrally than dorsally, laterodorsal angles roundly produced, caudal margin near base strongly produced posteriorly, in posterior view the pygofer with a single process on the midventral margin, lateroventral margins not well defined. Parameres widely divergent apically. Diaphragm of pygofer broad, dorsally produced and incised in middle. Suspensorium ring-like ventrally. Aedeagus tubular, not twisted at base, subapex bearing teeth on both sides. Anal segment deeply sunk into the dorsal emargination of pygofer, caudoventral angles each produced in a spinose process.

#### Remarks.

After being established by Motschulsky (1863), the genus *Mestus* was subsequently studied by Melichar (1903) and Distant (1906). However, the placement of this genus was unclear and was not treated in Muir’s phylogeny of the family Delphacidae because Muir did not agree with the original description of the type species (Muir 1915). Thereafter, Muir (1917) thought Melichar had confused *Anectopia mandane* Kirkaldy with *Mestus morio* Motschulsky, just as Fennah (1973–75: 85) stated: “he [Melichar] was wrong in interpreting *Anectopia mandane* Kirkaldy as *Mestus morio*. Motschulsky describes *Mestus morio* as having a strong median frontal carina, and his figure shows that the tegmina are not ornamented. *Anectopia mandane*, by contrast, has no median carina on the frons…”. The diagnosis of the type species, especially the male genital characters, became more identifiable after the work of Fennah (1973–75), Meanwhile, Fennah reconfirmed and treated *Mestus testaceus* Motschulsky and *Anectopia atrata* Muir as junior synonyms of *Mestus morio* Motschulsky, respectively. This study agrees with Fennah, who suggested *Anectopia atrata* Muir was a junior synonym of *Mestus morio* Motschulsky because the illustrations of *Anectopia atrata* (see Muir 1917, Figs 22, 22a, 22b) meet the definition of the genus *Mestus*.

The genus *Anectopia* Kirkaldy was established by Kirkaldy (1907). Muir (1915) checked its type species and placed this genus in the Delphacini of Delphacinae with two species (*Anectopia mandane* Kirkaldy, 1907 and *Anectopia igerna* Kirkaldy, 1907) known so far. Although *Anectopia* lacks a redescription after its establishment, the genus *Mestus* studied here differs from *Anectopia* in the post-tibial spur not having fine teeth along the posterior margin based on the works of Kirkaldy (1907), Muir (1915) and Fennah (1973–1975).

*Mestus* was once placed in Araeopini of the Araeopinae by Metcalf (1943); later it was assigned to the Tropidocephalini of the Delphacinae (Fennah 1973–75). This genus is currently recognized as a member of the Delphacini within Delphacinae (Asche 1985; Yang 1989; Ding 2006). From the keys of Yang (1989) and Ding (2006), the diagnosis of this Oriental genus is rather distinct and easily distinguished from other genera in the Delphacini by the post-tibial spur cultrate, solid, without teeth along posterior margin. Particularly in the key of Yang (1989), this genus is similar to two tropidocephaline genera: *Malaxa* Muir and *Tropidocephala* Stål. However, the post-tibial spur alone is not a sufficient indicator for tribal placement and for separating *Mestus* from other related genera, and there are many Delphacini that lack teeth along posterior margin (e.g., all of the former Alohini), features of the male genitalia are a better indication which should be considered for these genera. *Mestus* bears no obvious similarities with *Malaxa* or *Tropidocephala*. Furthermore, the composition and phylogeny of the Tropidocephalini needs to be reinvestigated.

Yang (1989) described *Mestus tungpuensis* based on “coleopterous” adults in Taiwan. According to the work of Bourgoin et al. (2015), the term coleopterous is useless to describe the tegmen precisely and has little morphological value. Therefore, the members of the genus *Mestus* have two wing forms, brachypterous and macropterous. The macropterous form of *Mestus* was described by Muir (1917) from the Philippines (*Anectopia atrata*, a synonym of *Mestus morio* as noted above). In the Chinese fauna, only the brachypterous form has been found so far. The wing polymorphism and biogeography of this genus need to be studied further.

#### Distribution.

China (Taiwan, Yunnan), Sri Lanka, Philippines.

### List of species and synonyms in *Mestus* Motschulsky

1. *Mestus morio* Motschulsky, 1863 synonyms: *Mestus testaceus* Motschulsky, 1863, synonymized by Melichar 1903: 105; *Anectopia atrata* Muir, 1917, synonymized by Fennah 1973–75: 85.
2. *Mestus tungpuensis* Yang, 1989

### Key to species of the genus *Mestus* (males)

**Table:** 

1	Medioventral process of pygofer widening in basal third then tapering to acuminate apex (Fennah 1973–75, Fig. 15); in posterior view the parameres lack teeth medially along inner margins (Fennah 1973–75, Figs 15, 16; Muir 1917, Figs 22, 22a)	***Mestus morio* Motschulsky**
–	Medioventral process of pygofer simple, not widening in basal third (Figs 8–11; Yang 1989, Figs 67G, F); in posterior view the parameres have distinct teeth medially along inner margins (Figs 8, 9, 12, 17; Yang, Figs 67D, L)	2
2	Male anal segment has two processes long, overlapped near bases (Figs 8, 18); caudoventral protrusion of pygofer near base well developed, subquadrate, extending to the same level as apex of medioventral process in profile (Fig. 9); aedeagus broadened in basal 1/3, ventral margin in profile almost straight medially (Figs 12, 14)	***Mestus cruciatus* sp. n.**
–	Male anal segment with two processes short and separated, not overlapped near base (Yang 1989, Fig. 67D), caudoventral protrusion of pygofer near base moderate, not extending to the same level as apex of medioventral process in profile (Yang 1989, Fig. 67E); aedeagus in profile slightly broadened in middle, ventral margin arched medially (Yang 1989, Fig. 67I)	***Mestus tungpuensis* Yang**

### Mestus cruciatus sp. n.

Taxon classificationAnimaliaHemipteraDelphacidae

http://zoobank.org/2FE82B05-6733-4BE2-A101-0A16AE495B1A

Figures 1–7 , 8–19

#### Description.

Brachypterous: Total length (from apex of vertex to the tip of abdomen): male (n=16) 2.40–2.75 mm, female (n=15) 2.65–2.88 mm; tegmina length: male (n=16) 1.85–1.90 mm, female (n=15) 1.88–1.98 mm.

**Figure 1–7.**
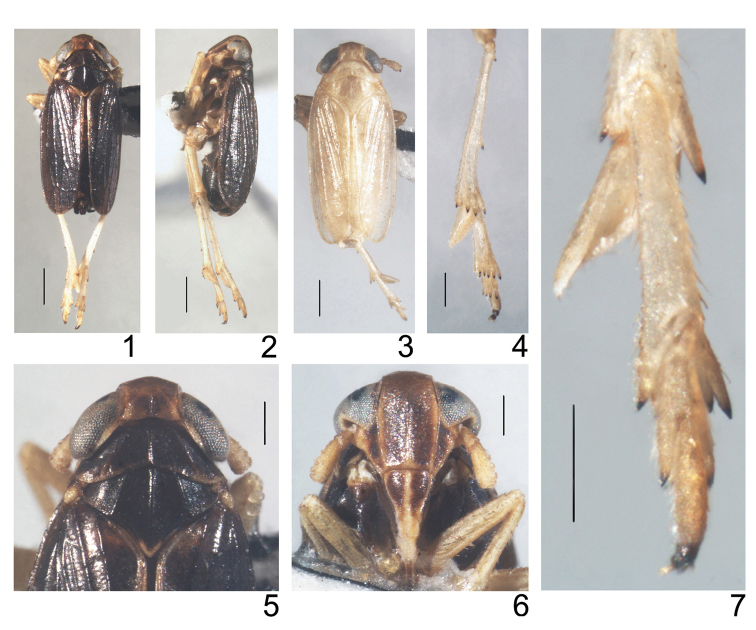
*Mestus cruciatus*, sp. n. **1** male adult, dorsal view **2** male adult, left lateral view **3** female adult, dorsal view **4** metatibia, metatarsus and post-tibial spur **5** head and thorax, dorsal view **6** frons and clypeus **7** metatarsus and post-tibial spur. Scale bars = 0.5 mm (Figs **1–3**); 0.2 mm (Figs **4–7**).

*Color*. General color of male dark brown (Figs 1, 2). Vertex, frons and genae blackish brown (Figs 1, 5, 6). Eyes grayish black (Figs 1, 2, 5, 6). Antennae pale brown (Figs 1, 2, 5, 6). Pronotum, mesonotum, tegmina and abdomen dark brown (Figs 1, 2, 5); in some specimens the posterior margin of pronotum and scutellum brown. Postclypeus blackish-brown except apex and median carina yellow (Fig. 6). Longitudinal veins of forewing speckled with black brown granules (Figs 1, 2, 19). Legs yellowish brown except fore- and middle coxae brown, apices of spines on tibiae and tarsi of hind legs black (Figs 4, 7). General color of female beige (Fig. 3). Tegmina semitransparent (Fig. 3). Ovipositor brown to blackish brown.

*Structure*. Vertex at about 1.32 times as broad as long in midline, slightly narrower at apex than at base (about 0.97: 1), anterior margin rounded, slightly projecting in front of eyes, lateral margins concave in dorsal view, submedian carinae originating from near 1/3 base of lateral carinae and feeble at apex (Figs 1, 5). Y-shaped carina with lateral arms faint, basal compartment shallowly concave, wider at base than greatest length (about 1.95:1) (Fig. 5). Fastigium rounded (Fig. 2). Frons longer in midline than maximum width about 1.61:1, widest at level of ocelli, lateral carinae slightly convex medially, median carina feeble at base (Fig. 6). Postclypeus wider at base than frons at apex (about 1.16:1), post- and anteclypeus together approximately 0.89× the length of frons (Fig. 6). Rostrum almost reaching meso-trochanters. Antennae terete, apex reaching to near the middle of postclypeus, scape longer than wide at apex (about 1.51:1), pedicle nearly twice the length of scape (Fig. 6).

Pronotum in midline slightly shorter than length of vertex (about 0.85:1), lateral carinae slightly curved, not reaching posterior margin of pronotum (Figs 1, 5), Mesonotum medially ca. 1.14 times longer than vertex and pronotum together, lateral carina almost straight, reaching posterior margin, median carina obscure before apex of scutellum (Figs 1, 5). Tegmina almost reaching or slightly surpassing apex of abdomen, longer than widest part about 2.48:1, widest near middle (Figs 1–3, 19). Spination of apex of hind leg 5 (3+2) (tibia), 7(5+2) (basitarsus) and 4 (2nd tarsomere) (Figs 4, 7). Hind tibiae 0.93–1.07 mm long, bearing 2 lateral teeth, post-tibial spur (0.33–0.38 mm) about 0.76× length of metabasitarsus, without identifiable teeth along posterior margin (Figs 4, 7).

**Figure 8–19.**
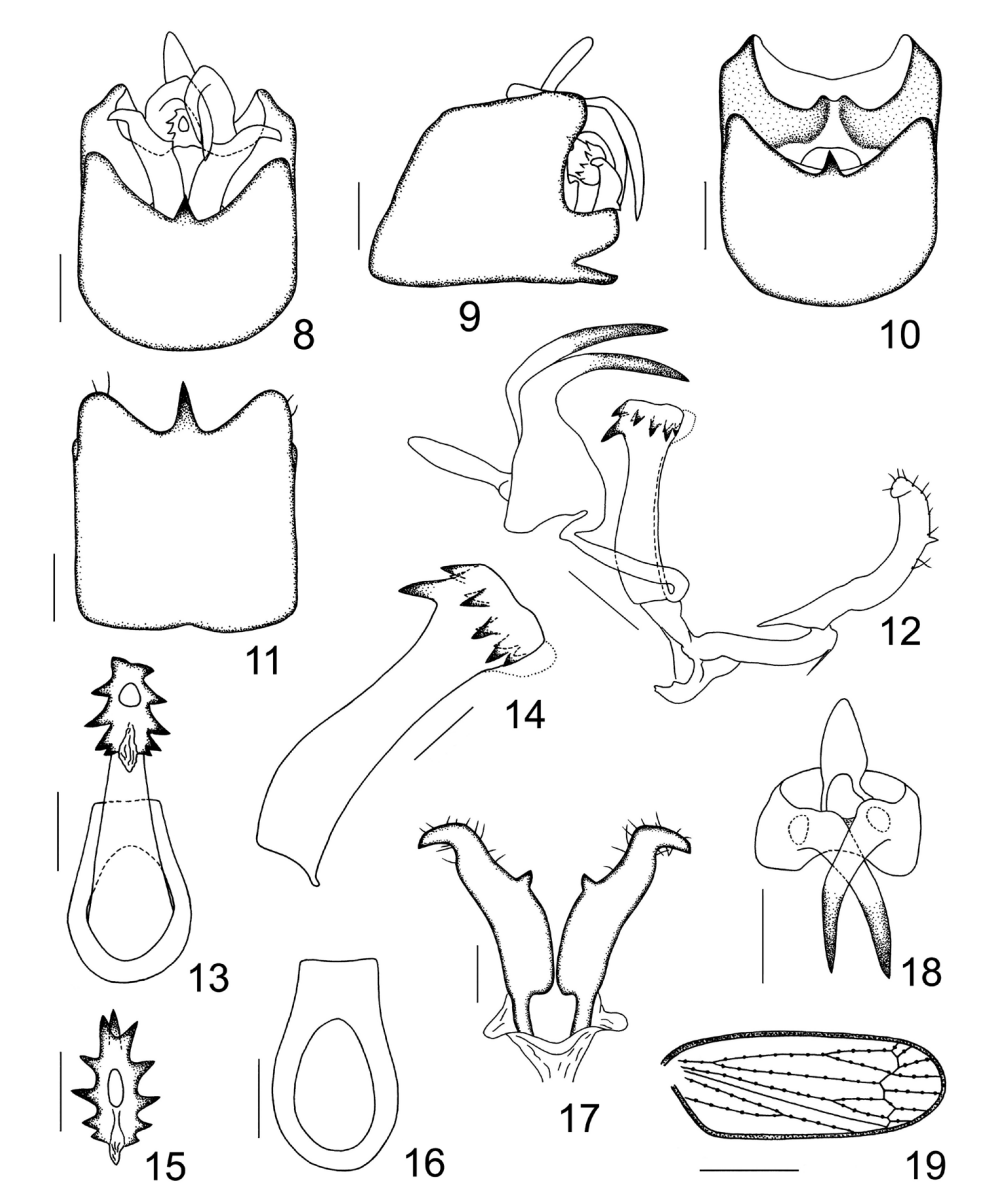
*Mestus cruciatus* sp. n. **8** male genitalia, posterior view **9** male genitalia, left lateral view **10** male pygofer, posterior view **11** male pygofer, ventral view **12** anal segment, aedeagal complex, connective and parameres, left lateral view **13** aedeagus, ventral view **14** aedeagus, left lateral view **15** apex of aedeagus, caudodorsal view **16** suspensorium, posterior view **17** parameres, posterior view **18** anal segment, caudodorsal view **19** tegmen. Scale bars = 0.2 mm (Figs **8–12**, **18**); 0.1 mm (Figs **13–17**); 0.5 mm (Fig. **19**).

*Male genitalia*. Pygofer in profile wider ventrally than dorsally, dorsolateral angle roundly produced, caudoventral margin near base with a well-developed, subquadrangular process, reaching the same level as medioventral process in profile (Fig. 9); in posterior view pygofer subquadrate, lateroventral margins excavated, medioventral process simple, spine-like in ventral view (Figs 8, 10, 11). Suspensorium ventrally ring-like, dorsally broad (Fig. 16). Dorsal margin of diaphragm produced, incised and membranous medially, in profile surpassing end of pygofer (Figs 9, 10). Parameres reaching the level of anal segment, sinuate, convergent at bases and then divergent distally, apices narrowed and strongly curved laterad, in posterior view each has a small tooth medially along inner margin (Figs 8, 9, 12, 17). Aedeagus moderate, in profile broadened dorsally in basal 1/3, ventral margin almost straight medially, at apex has a membranous tag on ventral side; in dorsocaudal view the aedeagus armed with approximately ten teeth circling the apical orifice, another bigger tooth, if present, shifted basally on the dorsal side (Figs 12–15). Male anal segment collar-shaped, laterocaudal margin with a long spinous process, overlapped near bases (Figs 8, 9, 12, 18).

#### Species examined.

Holotype. ♂ (brachypterous, NWAFU), China, Yunnan Province, Weixi County, 13-VIII-2010, coll. Meng Zhang. Paratypes. 15♂♂, 15♀♀ (brachypterous, NWAFU), same data as holotype.

#### Etymology.

This specific name alludes to the two overlapped processes near bases of the anal segment.

#### Host plant.

Unknown.

#### Discussion.

*Mestus cruciatus* sp. n. differs from *Mestus tungpuensis* Yang in having the caudoventral protrusion of pygofer near base well developed, extending to the same level as apex of medioventral process in profile; the aedeagus broadened dorsally in basal 1/3, ventral margin in profile almost straight medially. It differs from *Mestus morio* Motschulsky in having the medioventral process of pygofer simple, not widening in basal third; the inner margin of parameres each with tooth medially in posterior view. Furthermore, the new species differs from both species in having the lateroventral processes of male anal segment overlapped near bases.

#### Distribution.

Yunnan Province (in southwest China).

## Supplementary Material

XML Treatment for Mestus

XML Treatment for Mestus cruciatus
